# Protocol for quantifying the thickness and puncture resistance properties of solitary bee cocoons using *Osmia lignaria* as a model

**DOI:** 10.1016/j.xpro.2025.104191

**Published:** 2025-11-14

**Authors:** Oran Wasserman, Mallory R. Wootton, Spencer Fairbanks, Brianne E. Bell, Mary-Kate F. Williams, Justin A. Jones

**Affiliations:** 1 Department of Biology, Utah State University, Logan, UT, USA

**Keywords:** Biotechnology and bioengineering, environmental sciences, material sciences, protocols in Entomology, Special issue

## Abstract

Solitary bee cocoons serve as protective barriers against parasitoids, yet their puncture resistance properties remain understudied. Here, we present a protocol for measuring puncture resistance in *Osmia lignaria* cocoons using custom 3D-printed fixtures. We describe steps for determining bee sex, preparing cocoon segments, measuring segment thickness, testing puncture resistance using 27.5-gauge sharp needles on an MTS Synergie 100 mechanical testing instrument, and analyzing the data. The protocol generates quantitative data on puncture resistance properties and thickness measurements for comparative studies.

## Highlights

• Protocol for testing solitary bee cocoon puncture resistance properties
• Steps for measuring thickness in *Osmia lignaria* cocoons
• Instructions for quantifying puncture resistance properties in *Osmia lignaria* cocoons
• Custom 3D-printed fixtures for cutting, mounting, and testing cocoon segments

## Abstract (editor highlights)

Publisher’s note: Undertaking any experimental protocol requires adherence to local institutional guidelines for laboratory safety and ethics.

## Graphical abstract

**Figure:**
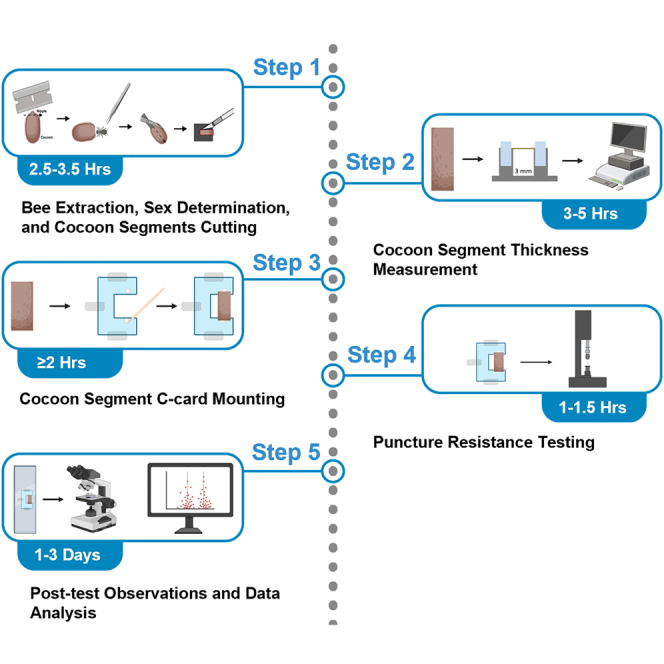

## Before you begin

Solitary bees, comprising approximately 75% of all bee species, are essential pollinators in agricultural and natural ecosystems.^1^^,^^2^ *Osmia lignaria* Say (Hymenoptera: Megachilidae) is a solitary bee, commonly known as the blue orchard mason bee, and is an important orchard pollinator in the western United States of America (USA).^3^^,^^4^^,^^5^ Some solitary bees, such as *O. lignaria*, construct a silk-based cocoon during development.^6^^,^^7^^,^^8^ The cocoon serves as a protective barrier during development, separating the prepupa from environmental stressors and parasitoid threats.^5^^,^^9^^,^^10^ Parasitic wasps, including *Monodontomerus* spp., target the pupa or prepupal stage by penetrating the cocoon with their ovipositors to inject eggs or paralyze the host.^5^^,^^11^^,^^12^ Parasitoid wasps use alternating ovipositor valve movements to reduce buckling risk during substrate penetration,^13^ with recent biomechanical analyses estimating maximum first valvula protraction forces of approximately 1.19 mN based on muscle cross-sectional areas and estimated muscle tension in *Diachasmimorpha longicaudata*.^14^ Despite the functional importance of solitary bee cocoons, many aspects remain understudied, such as the cocoon puncture resistance properties, especially compared to the extensively studied silkworm cocoons.^15^^,^^16^^,^^17^^,^^18^^,^^19^

### Innovation

Our protocol establishes reproducible methods for measuring cocoon thickness and testing puncture resistance properties using mechanical testing equipment and customizable 3D-printed fixtures. The approach includes a cocoon dimensional assessment using calipers, sex determination, segment preparation using a custom 3D-printed cutting stencil, thickness measurement with a CellScale MicroTester, and puncture testing using a sharp 27.5-gauge needle with 3D-designed mounts on an MTS Synergie 100 mechanical testing instrument. The protocol also describes the design considerations for the 3D-printed mounting and testing fixtures, serving as a blueprint for testing the cocoons of other solitary bees. Importantly, the testing parameters presented in this protocol are modified from established protocols for puncture testing of biological materials^20^ to provide the first puncture resistance properties testing of solitary bee cocoons, specifically *O. lignaria* cocoons. The C-card mounting protocol enables puncture testing in two directions (outer-to-inner and inner-to-outer layer) to analyze directional puncture resistance properties, as directional mechanical differences have been observed in layered biological materials.^20^^,^^21^ Our protocol generates quantitative data on cocoon thickness in millimeters (mm), maximum load in Newtons (N), maximum displacement in millimeters (mm), work to puncture in Newton-millimeters (N-mm), and stiffness in Newtons per millimeter (N/mm). Thickness measurements enable normalization of maximum load (N/mm) and work to puncture (N-mm/mm) values, accounting for variations between sample groups.^20^

### Institutional permissions

This protocol involves intact cocoons containing live *O. lignaria* adults that are collected prior to eclosion, followed by euthanizing the adults through incubation at −80°C and removal before processing the cocoon sample for thickness measurements and puncture resistance testing. No institutional permissions or ethics approvals are required for the puncture resistance testing procedures described. If collecting *O. lignaria* cocoons from wild populations, researchers should verify local and national regulations regarding insect collection in their specific geographic region, as requirements may vary by location.

### (Preparation one) Initial cocoon dimension assessment

**Timing: 3–5 min per cocoon**

This preparatory step determines the solitary bee cocoon dimensions to guide the design of cutting stencils, maximizing the number of segments obtainable from each cocoon. The cutting stencils facilitate the precise excision of multiple uniform rectangular pieces of cocoon using an X-ACTO knife.

1. Select 10–15 cocoons from your sample population.

***Note:*** Sample size should be carefully determined to ensure statistical adequacy, while accounting for potential constraints in obtaining solitary bee cocoons and the inherent mechanical variation in silk-based materials.^22^

2. For each cocoon, measure and record:
  a. Length along the longitudinal axis to the nearest 0.1 mm.
  b. Width was recorded using a dial caliper at three locations (middle, nipple end, and base end):
    i. Take two perpendicular width measurements at each location.
    ii. Record all measurements to the nearest 0.1 mm.
3. Record measurements in a Microsoft Excel spreadsheet with columns for cocoon ID, sex, length, and width measurements at each location.
4. Based on the collected data, design stencil dimensions based on measured size ranges, optimizing for maximum segment yield while ensuring adequate size for testing.

***Note:*** For *O. lignaria* cocoons, a 7 × 2 mm stencil was optimal based on the measured dimensions, yielding on average 5–7 segments per female cocoon and 3–4 segments per male cocoon. When adapting this protocol to different bee species, calculate the expected number of segments per cocoon based on species-specific dimensions to ensure adequate sample sizes.

### (Preparation two) 3D-printed fixture preparation

**Timing: 2–4 h for printing and assembly**

This preparatory step involves 3D printing the custom fixtures required for consistent cocoon segment cutting, mounting, and puncture testing. Figures S1–S5 provide diagrams with detailed information regarding the dimensions and specifications of each custom 3D-printed fixture used in this protocol.

5. Print the following 3D-printed fixtures:
  a. 3D-printed clamps that are used to clamp the cocoon segments onto the 3D-printed tower mount (Figure S1; STL file can be found in Data S1).
  b. 3D-printed tower mount used to determine cocoon thickness via the CellScale MicroTester (Figure S2; STL file can be found in Data S2).
  c. 3D-printed cutting stencil with 7 mm × 2 mm opening (Figure S3; STL file can be found in Data S3).
  d. 3D-printed bottom fixture with 6 mm × 5.5 mm opening to allow for adequate needle penetrations through the cocoon segment (Figure S4; STL file can be found in Data S4).
  e. 3D-printed top fixture for needle mounting with integrated Luer-Lock adapter (Figure S5; STL file can be found in Data S5).

***Note:*** Make any necessary adjustments to the 3D-printed fixture design for compatibility with your testing equipment (MTS Synergie 100 mechanical testing instrument clamps, needle type).

***Note:*** Print the 3D fixtures according to the printing parameters described in the materials and equipment setup section (below).

***Note:*** Conduct test runs following this protocol to identify if any modifications are needed for the 3D-printed fixtures.

**Figure 1:**
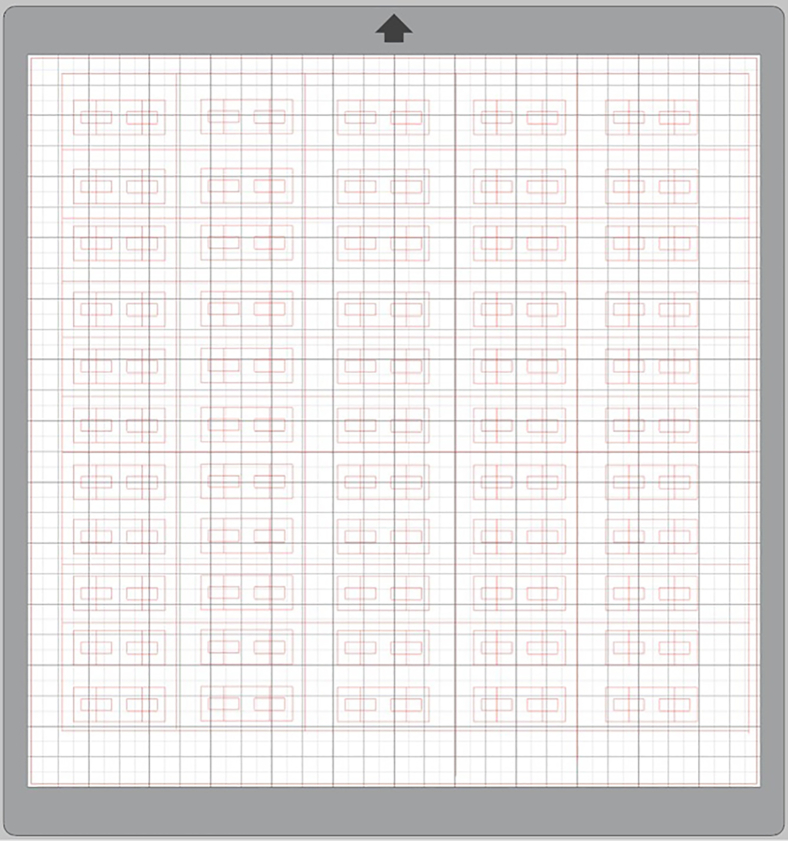
C-card template design A screenshot of the custom C-card template in Silhouette Studio 4.5 software showing the layout of the C-shaped templates (red outlines) arranged in a grid pattern for efficient cutting from the clear craft plastic sheets. Each C-shaped template features a 4.75 mm gap for mounting cocoon segments. The cutting mat grid provides a scale reference for template sizing.

## Key resources table

**Table:** 

REAGENT or RESOURCE	SOURCE	IDENTIFIER
**Biological samples**		

∼ Nine-month-old *O. lignaria* cocoons	USDA-ARS Pollinating Insects Research Unit, Logan, UT	N/A

**Chemicals, peptides, and recombinant proteins**		

95 % EtOH	Pharmco	Cat#111000190SS05

**Software and algorithms**		

MicroTester software (version 6.08)	CellScale Biomaterials Testing	https://www.cellscale.com/
PrusaSlicer (version 2.6.0)	Prusa Research	https://www.prusa3d.com/
Silhouette Studio (version 4.5)	Silhouette	https://www.silhouetteamerica.com/
TestWorks 4	MTS Systems Corporation	https://www.mts.com/
Motic Images Plus 2.0M	Motic	https://www.motic.com/
OriginPro 2025	OriginLab	https://www.originlab.com/
SolidWorks Education Edition 2023	Dassault Systèmes	https://www.solidworks.com/
Microsoft Excel	Microsoft	https://www.microsoft.com/
ImageJ	ImageJ	https://imagej.net/ij/

**Other**		

Single-edge razor blade	Razor Blade Company	Cat#66-0412
Mettler AE 100 Analytical Balance	Mettler Toledo	N/A
Dial Caliper	Bel-Art Products (ScienceWare)	Cat#13416-0001
Light microscope (Motic)	Motic Instruments USA Inc.	Model BA310 Digital LED Compound Microscope
Size 000 gelatin capsules	Capsule Depot	N/A
VWR Dissecting forceps, round tip	VWR	Cat#82027-394
VWR Dissecting scissors, sharp tip, 6^1^/_2_	VWR	Cat#82027-592
Loctite Super Glue Ultra Liquid Control 4g	Loctite	Cat#1647358
Digital microscope	Keyence Corporation of America	Digital microscope with VH-Z20W ultra-small high-performance zoom lens
Microscope slides	Globe Scientific Inc.	Cat# 1301, Plain Classic Microscope Slides
27.5-gauge sharp needles	Becton Dickinson	BD PrecisionGlide™ 27 G x 1/2 in, SKU: 305197
MicroTester G2	CellScale Biomaterials Testing	MTG2
Transparent tape	3M	UPC: 00076308872175
6″ Pocket Steel Ruler, 1/32″ & 1/64″ Graduations	Mitutoyo	Model #950-301
Samsill 12 × 12 × 0.007 inches clear craft plastic sheets	Samsill	B084HNGQPD
Silhouette Cameo 4	Silhouette	N/A
Type B AutoBlade	Silhouette	SILH-BLADE-AUTO-2
Kraft blade (3 mm)	Silhouette	SILH-BLADE-KRAFT-2
Cutting mat	Silhouette	N/A
Semi-tempered glass	Icona Bay on Amazon	B07QW19RQM
Gloss black spray paint + primer	Rust-oleum	334026
MTS Synergie 100 mechanical testing instrument with a 50N load cell	MTS Systems Corporation	N/A
Wood toothpick 4 inches (in)	N/A	N/A
X-ACTO 2 Knife With Safety Cap	X-Acto	UPC: 079946236026
PLA	OVERTURE3D	CAT #63646B
Original Prusa i3 MK3S	Prusa Research	N/A
Laboratory label tape	Bartovation through Amazon	B0889LYH61
RH and temperature device	AcuRite	Digital Humidity and Temperature Monitor

***Alternatives:*** The specific equipment and software described in this protocol can be replaced with equivalent alternatives from other manufacturers and sources.

## Materials and equipment

### 3D printing parameters for custom fixtures

• Printer: Original Prusa i3 Mk3.
• Filament: 1.75 mm Overture PLA.
• Slicing Software: Prusa Slicer 2.6.0.
• 3D printing settings:
  ○ Layer height: 0.15 mm.
  ○ Supports: On build plate only.
  ○ Brim: 5 mm.
  ○ Infill: 100%.
  ○ Rectilinear fill pattern.

### Cleaning and assembly

• Remove burrs and overhangs with a single-edge razor blade before use.
• Clean all components using 70% EtOH before the mounting and testing procedures.

### Alternative materials

• PLA can be substituted with PETG for increased durability.
• If a Prusa printer is not available, any FDM printer with ≥0.15 mm resolution may be used.

### Specimen collection and storage

• Collect cocoons from natural or managed populations following local regulations.
• Store cocoons in gelatin capsules to maintain specimen integrity and facilitate handling.
  ○ This study employs commercially available large gelatin pill capsules (26.1 mm length, size 000) to house cocoons.
• Maintain consistent temperature (20°C–21°C) and relative humidity (14%–30%) when storing, prepping, and testing the cocoons.
• Document collection location, date, storage conditions, and cocoon age for each batch.
• Allow cocoons to acclimate to testing conditions for 24–48 h before use.

***Note:*** For this study, *O. lignaria* cocoons were provided by the USDA-ARS Pollinating Insects Research Unit in Logan, UT. Cocoons used in this study were offspring from bees that were released in Zollinger’s apple orchard in Providence, UT. Parents were released in the apple orchard on 6 May 2024. Nests were collected every 3 days from 10 May to 24 May 2024 and brought into lab conditions to develop (∼21°C). Bees in cocoons were stored at 4°C during the winter, 1 October 2024 – Spring 2025. Before spring emergence, bees in cocoons were removed from their natal nest and transferred to gel capsules. Cocoons in gelatin capsules were stored at −80°C. Prior to use in this protocol, cocoons were equilibrated to ∼21°C.

### C-card preparation

• C-cards are prepared from clear craft plastic sheets using a Silhouette Cameo 4.
• Rinse the outer layer of clear craft plastic sheets with warm water and trim to 31 × 31 centimeters (cm) to fit the cutting mat.
• Use Type B AutoBlade (set to 10) and 3 mm Kraft Blade (set to 15–20) for cutting.
• Execute 2–4 passes with the first blade and 6 passes with the second blade using the custom design in Silhouette Studio 4.5 (Figure 1).
• C-cards feature a 4.75 mm gap for mounting the cocoon segment.
• Complete C-card preparation protocol available in Wasserman (2025).^8^

***Note:*** C-cards are specialized mounting frames cut from clear plastic sheets that secure cocoon segments for puncture testing. The C-shaped design features a central gap (4.75 mm) that allows needle penetration while providing support and standardizing the span length across which specimens are tested.

### Glass cutting mat preparation

• The glass cutting mats are prepared using two panes of semi-tempered glass, black spray paint, and laboratory label tape.
• Wash the two panes of glass with soap and water to remove dust from shipping or storage.
• In a well-ventilated area, apply an even coat of black spray paint to one side of each pane (other high contrast colors work too).
• Once the paint is dry, inspect and add another coat, repeating the process until there aren’t any bare spots.
• Allow the panes to dry fully, then carefully align both panes with the paint on the inside (alternatively, you can spray just one pane instead of both).
• Using laboratory label tape, secure the two panes together (ensure the paint is on the inside of the stack of panes) by taping along all of the edges of the panes.

## Step-by-step method details

### Bee extraction, sex determination, and cocoon segment cutting

**Timing: 2.5–3.5 h for 10 cocoons (30–50 cocoon segments)**

This major step involves cutting the intact cocoon to extract the bee specimen, determining sex based on morphological characteristics, and excising uniform cocoon segments using a custom 3D-printed stencil. Sex determination is important to account for potential differences in physical and puncture resistance properties between sexes, as differences in cocoon sizes have been observed in *Osmia* species.^23^

1. Place the cocoon on a black glass mat to provide contrast during dissection.
2. Using a razor blade, carefully cut and remove the nipple (cap) from one end of the cocoon. Discard the nipple.
3. Using dissecting scissors, make a longitudinal cut along the length of the cocoon to create an opening that allows access to the adult bee inside.
4. Carefully extract the adult bee from the cocoon using dissecting forceps.
5. While holding the bee with dissecting forceps, examine both the bee and the cocoon for any signs of parasitoid, bacterial, or fungal infection.

**CRITICAL:** If any evidence for parasitoid, bacterial, or fungal infection is observed on the cocoon or excised bee, both the bee and the cocoon should be disposed of appropriately. Information regarding infection identification methods for *Osmia* species can be found in LeCroy et al. (2023) and Hedtke et al. (2015) for fungal pathogens.^24^^,^^25^ However, additional resources may be needed for the comprehensive identification of parasitoid and bacterial infections.

6. Observe the bee’s morphological features to determine sex while holding it with dissecting forceps.

***Note:*** Key identifying features for *O. lignaria* include:

**Males:** Metallic dark blue to blue-green coloration, distinct white hair patch on the face, and longer antennae relative to females.^5^

**Females:** Larger and more stout than males, larger mandibles, same metallic coloration as males but lacking extensive white facial hair, shorter antennae relative to males, and presence of horn-like prongs on the lower face visible under magnification.^5^^,^^26^

**Additional distinguishing features:** Females possess a scopa (brush of long hairs) located under the abdomen for pollen collection, while males lack this structure.^5^

**Figure 2:**
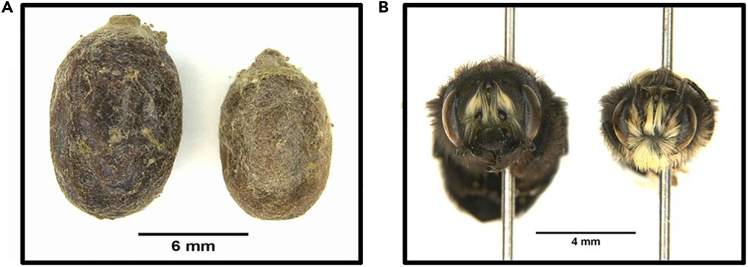
*Osmia lignaria* cocoon morphology and sex identification (A) Intact cocoons showing size variation between female (left) and male (right) specimens. Scale bar = 6 mm. (B) Adult bees extracted from the intact cocoons during sex determination (protocol steps 6–9), showing female (left) and male (right) facial characteristics used for morphological identification. Scale bar = 4 mm. Pictures were taken via Keyence VHX500FE microscope.

7. For specimens that require closer inspection due to the size of the bee or potential mechanical damage to the bee, pin the adult bee and examine it under a microscope for more precise morphological analysis.
  a. An example of a male and female pinned specimen can be found in Figure 2.
8. Record the sex determination and any relevant observations about the specimen’s condition in your data sheet.
9. Place the two halves of the previously excised cocoon onto a black glass surface.

***Note:*** Any surface that provides enough contrast and can be easily cleaned can be used. However, tempered glass is preferable because it is less prone to scratches and shattering while being easy to clean.

10. Cut the halved cocoons into quarters in the longitudinal direction, using a ruler and dissecting scissors. See solutions to potential pitfalls under troubleshooting 1.

***Note:*** Cutting the cocoon into quarters helps reduce the curving of the sections while placing the 3D stencil over them.

11. Position a 3D-printed stencil over a quarter cocoon section to define the segment area.
  a. Align the stencil consistently between cocoon samples (e.g., parallel to the longitudinal axis of the cocoon).
12. Use an X-ACTO knife to cut out the cocoon segment along the stencil edges.
13. Gently transfer the excised segments to a pre-labeled gelatin capsule used to store the same cocoon.

**CRITICAL:** Avoid damaging the cocoon segments or stretching them to minimize alterations in thickness or puncture resistance properties.

### Cocoon segment thickness measurement

**Timing: 3–5 h for 10 cocoons (200 thickness measurements)**

Robust thickness measurement is essential for normalizing stress and work values to accommodate the thickness variability in the cocoons, as was done with other biological samples.^20^ This step utilizes the CellScale MicroTester and custom-designed 3D-printed mounting towers to determine thickness.

14. Select the number of samples that will be utilized for thickness measurements per cocoon.

***Note:*** We selected one section per cocoon for thickness measurements, while the other segments were designated for puncture testing.

15. Power on the CellScale MicroTester and set up the image conditions that will provide a clear visualization of the cocoon segment.

***Note:*** To ensure consistency, use the same pre-determined setting across all cocoon segments.

16. Cut the 7 mm × 2 mm cocoon segment using an X-ACTO knife in the center to a width of 1 mm.

***Note:*** Due to the small size of the cocoon and its natural curve, the 1 mm segment will appear flat on the tower mount, allowing for an accurate measurement of its thickness.

**Figure 3:**
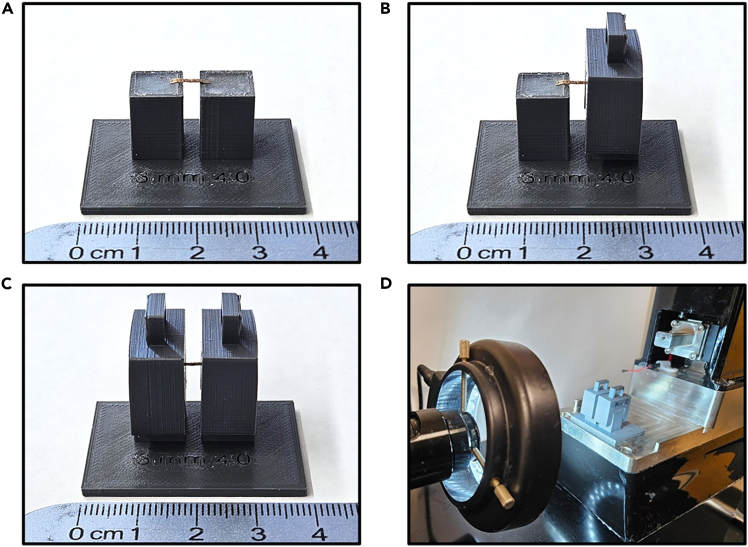
Custom 3D-printed tower mounts for cocoon segment thickness measurement (A) The cocoon segment is placed perpendicular to the tower mount. (B) First clamp applied, ensuring the cocoon is not stretched and remains perpendicular to the tower mount. (C) The second clamp is applied to secure the specimen. (D) The tower mount is positioned at the edge of the Test Chamber with the fluid bath removed, directly positioned in front of the CellScale MicroTester camera.

17. Place the cocoon segment perpendicular to the tower mount (Figure 3A).
18. Carefully apply the first clamp, ensuring the cocoon is not stretched and remains perpendicular to the tower mount (Figure 3B).
19. Apply the second clamp and place the tower mount at the edge of the CellScale MicroTester test chamber, with the fluid bath component removed (Figure 3D).

***Note:*** Both the test chamber and the fluid bath component are part of the CellScale MicroTester testing apparatus. More information regarding the components of the apparatus can be found in the manufacturer's manual.

**Figure 4:**
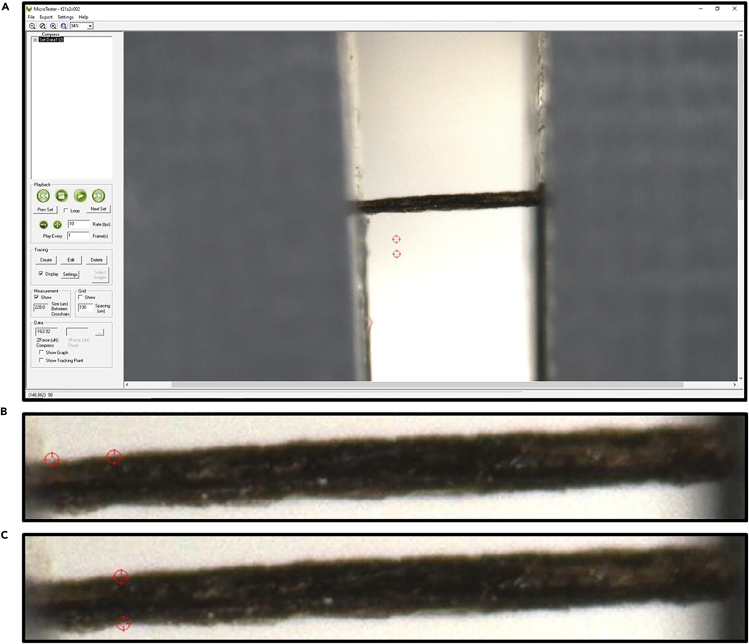
Thickness measurement workflow using CellScale MicroTester software (A) MicroTester software interface showing a mounted cocoon segment positioned for measurement with measurement crosshairs visible (red circles). (B) Close-up view demonstrating crosshair positioning approximately 250 μm from the tower mount edge for the first measurement point. (C) Crosshairs are positioned perpendicular to the specimen length to capture the thickness measurement at the designated location. Measurements are taken at 500 μm intervals along the specimen length as described in the protocol.

20. Adjust the camera position until the mounted cocoon segment is positioned directly in front of the camera and a clear, focused view of the specimen is achieved (Figure 3D).
21. Start a test recording to generate a test file, run for a few seconds to ensure file formation, and then stop the test.
22. Click on the “Analyze and Review Images” function in the software to open the test file and access the measurement interface with crosshairs (Figure 4A).
23. Using the crosshairs tool, take a measurement approximately 250 micrometers (μm) from one end of the mounted specimen (Figure 4B).
24. Place the crosshairs tool perpendicular across the cocoon segment and record the measurement (Figure 4C).
25. Move the crosshairs tool at 500 μm intervals across the specimen and record a total of five thickness measurements. See potential pitfalls and solutions under troubleshooting 2.
26. Rotate the mounted tower apparatus 180 degrees to record thickness measurements from the other side of the 1 mm segment.
27. Re-adjust the camera and repeat steps 26 and 27.
  a. Remove the cocoon segment from the tower mount and discard it once measurements have been taken for both viewpoints of a single section.
28. Clamp down a new segment and repeat steps 17–28.

***Note:*** From each original cocoon segment (7 × 2 mm), 20 thickness measurements are collected.

***Note:*** Thickness measurements can alternatively be performed using a light microscope. However, modifications will be needed to mount cocoon samples with minimal handling, and initial testing showed this approach to be less reliable than the MicroTester approach.

### Cocoon segment C-card mounting

**Timing: ≥2 h for 10–15 samples**

This step allows for the mounting of the cocoon segments onto clear craft plastic cut C-cards. The mounted C-cards can then be mounted onto the 3D-printed fixtures for the cocoon puncture.

29. Use custom-cut C-cards with a 4.75 mm gap designed based on the cocoon segments obtained from the 3D-designed stencil.
30. Tape each side of the C-card onto a microscope slide with transparent tape (∼2 mm x 5 mm).
  a. Ensure the transparent tape doesn’t cover the space near the gap where the cocoon segment will be mounted.
31. Carefully apply transparent tape to one end of the cocoon segment and place the cocoon segment perpendicular onto the C-card.
32. Gently press the other end of the cocoon to the C-card surface and apply transparent tape to secure the cocoon segment to the C-card.

***Note:*** Ensure that the cocoon segment is pulled taut but not stretched across the C-card. This will aid in obtaining clearer puncture pictures using the microscope later and increase the reproducibility of the data acquired. Slack in the cocoon segments will alter the data acquired from the tests.

33. Using a needle or toothpick, apply a small amount of superglue to each end of the cocoon segment to secure it to the C-card.
34. Apply superglue to each arm of a second C-card at the exact locations on the first C-card.
35. Using dissecting forceps, carefully place the second C-card, superglue-side down, atop the C-card holding the cocoon segment.
36. Ensure that the C-cards are aligned and that the superglue is in contact with each end of the cocoon segment.
37. Allow the double-layered C-card mount to cure overnight. See potential problems and solutions for this process in troubleshooting 3.

**CRITICAL:** The double C-card system prevents cocoon segment detachment during puncture testing, as single C-card mounting may result in adhesive failure before cocoon puncture occurs.

### Puncture resistance testing

**Timing: 1–1.5 h for 10–15 samples**

This major step generates load (N) and displacement (mm) data by penetrating cocoon segments with a standardized needle under controlled conditions.

38. Turn on the MTS Synergie 100 mechanical testing instrument and open the MTS TestWorks software.
39. Set up the testing parameters in the MTS TestWorks software in compression settings.
  a. In our protocol, we set the cross-head speed at 5 mm/min.^20^
40. Mount a single-use, 27.5-gauge sharp needle to the top fixture via a Luer-lock adapter.
  a. Replace the needle after the end of each test.
41. Determine the direction of puncture for that sample group.

***Note:*** The double C-card mount allows for puncture testing in two different directions. Either outer layer to inner layer (labeled as up-down direction), or inner layer to outer layer (labeled as bottom-up direction).

**Figure 5:**
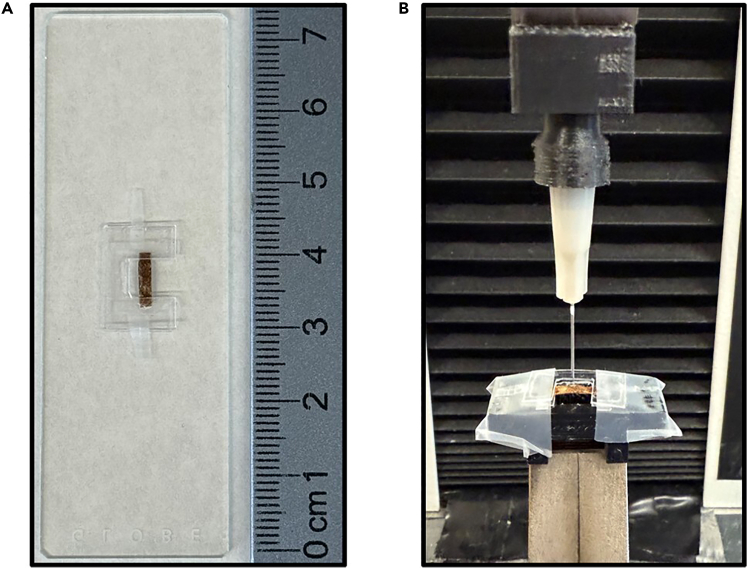
Puncture resistance testing setup (A) Cocoon segment mounted on C-card with transparent tape positioning for testing. (B) Set-up of the testing apparatus prior to testing with 27.5-gauge sharp needle aligned above mounted cocoon segment using custom 3D-printed fixtures on MTS Synergie 100 mechanical testing instrument.

42. Affix the cocoon-mounted C-card (Figure 5A) to the bottom fixture using transparent tape, positioning it horizontally over the 6 mm × 5.5 mm opening.
  a. Tape the C-card edges to the 3D-printed fixture surface to prevent slipping during testing.
43. Adjust vertical alignment so the needle is centered above the cocoon segment midpoint (Figure 5B).
44. Begin the test and record data until a sharp drop in the load (N) values is observed, accompanied by a visual observation of a puncture.

**CRITICAL:** Use a new needle for each test to minimize variability caused by the tip dulling.

45. End the test and export the raw load (N) and displacement (mm) data.

***Note:*** An example of steps 44–45 can be seen in Methods Video S1.

***Note:*** While an MTS Synergie 100 mechanical testing frame with a 50 N load cell was used here, any mechanical testing frame with an appropriately sized load cell can be used. The testing frame must be capable of performing compressive testing and have interchangeable clamps for testing compatibility with the 3D-printed fixtures used in this protocol.

Methods Video S1. Puncture resistance test of mounted cocoon segment via MTS Synergie 100, related to steps 44–45 and Figure 2B

### Post-puncture verification and data analysis

**Timing: 1–3 days (depending on sample size)**

This step involves microscopic examination of puncture sites to confirm complete needle penetration through the cocoon layers and verify proper test execution, followed by analysis of the data exported from the MTS Synergie 100 mechanical testing instrument after puncture testing.

46. After the puncture test, use transparent tape to tape the mounted C-card back onto a microscope slide.
47. Place the microscope slide under a light microscope.
48. Inspect the puncture site at different magnifications and record whether complete penetration occurred.
  a. Confirm that the needle created a clean puncture hole with no evidence of incomplete penetration or lateral tearing.
49. Capture digital images of puncture sites using the Motic microscope software to document complete needle penetration and puncture morphology.

***Note:*** Images should be saved for documentation and clearly demonstrate the puncture hole and confirm successful needle penetration through the cocoon material. Photograph representative examples for documentation.

**CRITICAL:** Only include test data from specimens showing complete needle penetration. Discard data from tests where puncture verification reveals incomplete penetration or excessive material damage.

50. Export the raw load (N) displacement (mm) data files from MTS TestWorks software as.txt files.

**CRITICAL:** Use consistent units throughout analysis and verify calculations using representative samples before processing the entire dataset.

51. Import the data into a pre-formatted Microsoft Excel template using the “comma” delimiter to correctly separate data into columns.^8^
  a. Detailed data import procedures are described in Wasserman et al., 2025.^8^

**CRITICAL:** Ensure consistent file naming conventions that include sample ID, test date, and group designation.

52. Adjacent to the raw data imported from MTS TestWorks software, create the following column headers: zeroed displacement (mm), zeroed load (N), maximum load (N), maximum displacement (mm), stiffness (N/mm), work to puncture (N-mm), normalized load (N/mm), and normalized work to puncture (N-mm/mm).
53. Calculate primary puncture resistance parameters for each cocoon segment using the following equations:
  a. Zeroing out the displacement: *D*_*actual*_ = *D*_*atpoint*_ − *D*_*startingvalue*_
    i. Where *D*_*starting value*_ is the point where data logging begins (before needle contact), and *D*_*at point*_ is the current displacement value.
  b. Zeroing out the force load: *F*_*actual*_ = *F*_*atpoint*_ − *F*_*startingvalue*_
    i. Where *F*_*starting value*_ is the baseline force load reading before needle contact, and *F*_*at point*_ is the current force value. Force load is referred to as load (N) in the text.
54. Observe the raw load (N) and displacements (mm) column and identify when the load (N) values start to increase consistently.
55. Select the point that will represent where you will start to analyze the data.

***Note:*** If issues arise in identifying where the test begins in the raw data, you can plot the raw load (N) and displacements (mm) and either identify the point on the graph or compare your plot to the one in the testing apparatus for reference.

56. Place the zeroing formulas (step 54a, b) and apply the formulas until a sharp drop in the load (N) is detected and the values are near zero.

***Note:*** It is recommended to plot the raw load (N) and displacement (mm) as previously discussed and identify when the test ends.

57. Calculate primary puncture resistance parameters using the following equations:
  a. Maximum load: *F*_*peak*_ = *max*⁡(*F*_*actual*_)
    i. Record the maximum load (N) value from the zeroed load column.
  b. Maximum displacement: *D*_*peak*_ = *max*⁡(*D*_*actual*_)
    i. Record the maximum displacement (mm) value from the zeroed displacement column.
  c. Stiffness: *S*_*puncture*_ = Δ*D*_*load*_ / Δ*D*_*displacement*_
    i. Calculate the stiffness (N/mm) by applying the slope of the initial linear region of the load (N) - displacement (mm) curve.
    ii. To ensure consistency, select 20 data points for each sample.
  d. Work to Puncture: $W_{puncture\;}=\int_{0}^{Dpuncture}F\left(D\right)\;dD$
    i. Calculate the work to puncture (N-mm/mm) by calculating the area under the load (N) - displacement (mm) curve using trapezoidal integration.
  e. Normalized load: *F*_*normalized*_ = *t*_*mean*_ / *F*_*peak*_
    i. Where t_mean_ is the mean segment thickness (mm) per either female or male.
  f. Normalized Work to Puncture: *W*_*normalized*_ = *t*_*mean*_ / *W*_*puncture*_
    i. Where t_mean_ is the mean segment thickness (mm) per either female or male.
58. Copy and paste the load and displacement columns as links into a separate Microsoft Excel tab to generate load-displacement curves for each specimen.
  a. Create graphs with displacement (mm) on the x-axis and load (N) on the y-axis.
  b. If noise is present in the load (N) - displacement (mm) curves, apply a 5-point moving average to load (N) and displacement (mm) values to reduce variability likely due to the testing parameters and not the sample.^8^ See helpful pointers in troubleshooting 4.
59. Create a summary table for each test group containing all samples from that group.
  a. Include all sample measurements: maximum load (N), maximum displacement (mm), stiffness (N/mm), work to puncture, normalized maximum load (N/mm), and normalized work to puncture(N-mm/mm).
  b. Ensure consistent units throughout the dataset.
60. For each test group table, calculate the average and standard deviation for each property using Microsoft Excel functions.
  a. Use Microsoft Excel function = *AVERAGE*(*columnX*) for mean values.
  b. Use Microsoft Excel function = *STDEV*(*columnX*) for standard deviation values.
61. Combine the averaged properties and standard deviations in a final summary table containing values from all test groups.
  a. Include columns for test group ID, mean values, and standard deviations for each measured property.
  b. This table should contain one row per test group with summary statistics.

**CRITICAL:** Verify all calculations using representative samples before processing the entire dataset.

## Expected outcomes

This protocol generates quantitative puncture resistance properties data and physical measurements that characterize cocoon puncture resistance, enabling statistical comparison between experimental groups. Researchers can expect to complete datasets for n = 10–15 specimens per group within 5 days, depending on sample availability and testing schedule.

Initial cocoon dimensional measurements guide the design of 3D-printed mounting and testing fixtures to maximize sample yield from each cocoon. The summary of the initial measurements taken from the cocoons is in Table 1.

**Table 1:** Summary of initial cocoon measurements collected for the design of the 3D-printed cutting and mounting fixtures

Sex	Cocoon length (mm)	Mid-section diameter (mm)	Nipple section diameter (mm)	End section (mm)
Female	12.53 ± 0.64	6.61 ± 0.39	1.63 ± 0.25	5.77 ± 0.53
Male	9.42 ± 0.82	4.98 ± 0.61	1.46 ± 0.3	4.00 ± 0.53

Thickness measurements are crucial for normalizing puncture resistance properties across samples and evaluating the physical properties of the cocoons. For *O. lignaria* cocoons, this protocol yields segment thickness ranging from 0.08 to 0.28 mm for female cocoons, and 0.12–0.25 mm for male cocoons (Table 2). The measurement approach captures thickness variation across the sample’s surface using five measurements per view on two 1 mm sections, yielding 20 total measurements per original 7 × 2 mm segment. Researchers can expect to observe differences between male and female cocoons, supporting the importance of sex-based grouping in experimental design for comparative studies. Visual inspection during thickness measurement also helps identify potentially damaged segments that should be replaced before proceeding to puncture testing.

**Table 2:** Summary of cocoon segment thickness measurements

Sex	Mean thickness ± SD (mm)	Thickness range (mm)
Female	0.155 ± 0.043	0.08–0.28
Male	0.196 ± 0.035	0.12–0.25

**Figure 6:**
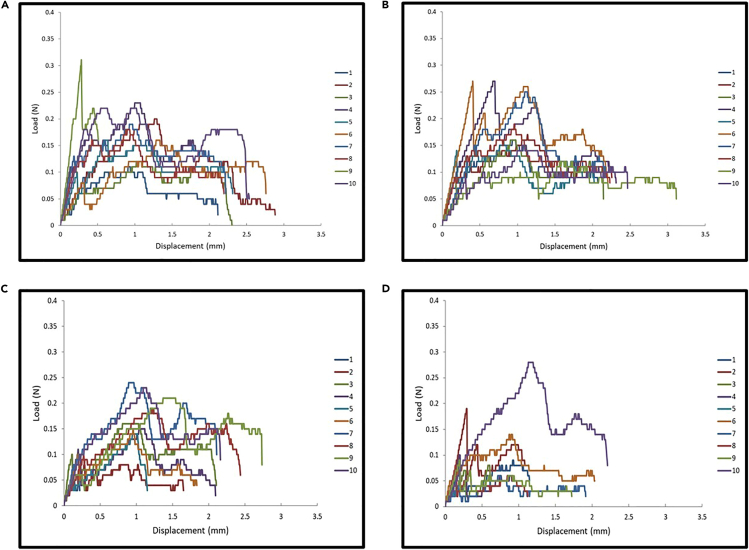
Representative load (N) - displacement (mm) curves from cocoon puncture testing using 27.5-gauge needles (A) Female cocoon segments tested in the top-down direction. (B) Female cocoon segments were tested in the bottom-up direction. (C) Male cocoon segments tested in the top-down direction. (D) Male cocoon segments were tested in the bottom-up direction. Each colored line represents an individual cocoon segment test (n = 10 per group).

Puncture resistance testing generates quantitative mechanical parameters that allow for characterizing the puncture resistance properties of *O. lignaria* cocoons (Table 2). *Osmia lignaria* cocoons were tested in both top-down (labeled as up) and bottom-up directions (labeled as down). Researchers can expect to observe puncture resistance properties variation between male and female cocoons, as well as between puncture directions (Table 3), which supports the importance of sex-based grouping and directional testing in experimental design for future comparative studies. Representative load (N) - displacement (mm) curves illustrate the typical responses to puncture loading, with observable patterns of data variability across sample test groups (Figure 6). Statistical analysis methods are described in the quantification and statistical analysis section.

**Table 3:** Summary of puncture resistance properties of *O. lignaria* cocoon segments by sex and testing direction (*n* = 10 per test group)

Test group ID	Max load (N)	Max displacement (mm)	Stiffness (N/mm)	Work to puncture (N-mm)	Normalized load (N/mm)	Normalized work (N-mm/mm)
Female Up	0.19 ± 0.06	2.18 ± 0.65	0.48 ± 0.28	0.25 ± 0.08	1.21 ± 0.37	1.59 ± 0.55
Female Down	0.19 ± 0.05	2.30 ± 0.31	0.44 ± 0.20	0.27± 0.06	1.23 ± 0.35	1.73 ± 0.37
Male Up	0.16 ± 0.06	1.85 ± 0.72	0.49 ± 0.17	0.20 ± 0.12	0.82 ± 0.31	1.03 ± 0.62
Male Down	0.12 ± 0.07	1.30 ± 0.65	0.35 ± 0.15	0.09 ± 0.10	0.59 ± 0.36	0.47 ± 0.52

**Figure 7:**
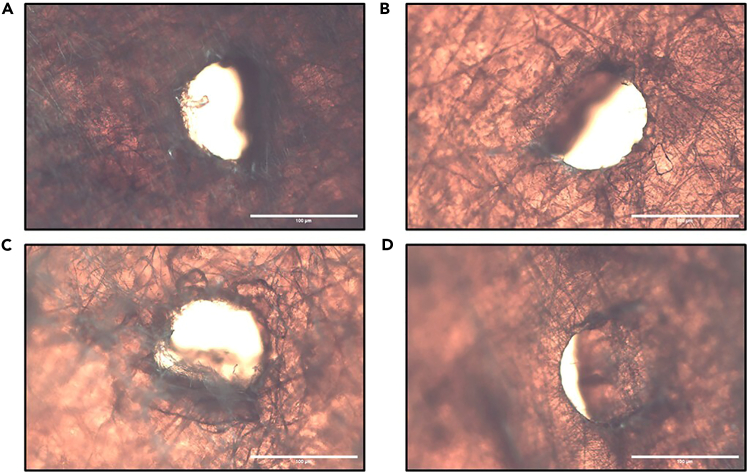
Post-puncture verification of cocoon segments Representative puncture sites following puncture testing with a 27.5-gauge sharp needle. (A) Female cocoon, top-down direction. (B) Female cocoon, bottom-up direction. (C) Male cocoon, top-down direction. (D) Male cocoon, bottom-up direction. Scale bars = 100 μm. Pictures were taken with the Motic light microscope at 100× total magnification. Scale bars were added via ImageJ.

Microscopic examination confirms complete needle penetration and validates proper test execution. Representative puncture sites exhibit clean, well-defined holes demonstrating successful penetration through all cocoon layers (Figure 7). Visual inspection aids in identifying complete penetration without peripheral material damage and ensures clear hole formation extending through the entire cocoon thickness. If incorrect or inconsistent penetration angles are recorded, refer to troubleshooting 5. This verification step maintains data quality by confirming that only valid mechanical testing results are included in subsequent analysis.

***Note:*** For more detailed morphological analysis of puncture sites and effects on the cocoon's microstructure, higher resolution techniques such as scanning electron microscopy (SEM) may be considered.

## Quantification and statistical analysis

The statistical analyses were performed using Microsoft Excel and OriginPro 2025 software (OriginLab Corporation). Data are presented in Tables 1, 2, 3 as mean ± standard deviation unless otherwise specified. Statistical significance was set at α = 0.05 for all tests. Thickness measurements were averaged across 20 measurement points per segment (n = 6 cocoons per sex). Normal distribution was assessed using the Shapiro-Wilk test, and statistical differences between sexes were analyzed using one-way ANOVA after confirming variance homogeneity with Levene’s test.

For mechanical testing data, normal distribution was assessed using the Shapiro-Wilk test for each experimental group combination (Female-Up, Female-Down, Male-Up, Male-Down). The sample size is n = 10 cocoon segments per experimental group for a total of 40 specimens tested. Due to the non-normal distribution detected in some groups (p < 0.05), the puncture resistance properties were analyzed using the non-parametric Kruskal-Wallis test, comparing all four experimental groups. Six mechanical properties were analyzed: maximum load (N), maximum displacement (mm), stiffness (N/mm), work to puncture (N-mm), normalized load (N/mm), and normalized work to puncture (N-mm/mm). When significant differences were detected (p < 0.05), pairwise comparisons between groups were conducted using Dunn’s post-hoc test. The raw data supporting the results presented in this study are available from the corresponding author upon request.

## Limitations

Several limitations should be considered when designing an experiment utilizing this protocol and interpreting results, specifically with other solitary bee species cocoons.

Silk-based materials such as *O. lignaria* cocoons^8^ exhibit high natural variability in thickness, mechanical, and compression properties across species.^21^^,^^27^^,^^28^ Careful considerations of sample size, processing conditions, and test parameters should be taken into account. While silkworm cocoons have been shown to exhibit layer-specific puncture resistance properties,^17^^,^^18^ the extent to which these findings apply to solitary bee cocoon remains to be determined through a comprehensive investigation using protocols such as the one described here. In addition, while we provide a dataset regarding approximately nine-month-old cocoons, future studies should investigate the relationship between the age and hydration state of the cocoon and the resulting puncture resistance properties, as hydration state has been reported to correlate with alterations in mechanical performance.^29^^,^^30^

Another point to consider is that the protocol requires specialized equipment (MTS Synergie 100 mechanical testing instrument, CellScale MicroTester, 3D-printed fixtures) that may not be available in all laboratories. Alternative testing approaches may be necessary when standard equipment is unavailable, though modifications should be validated against established protocols. Normalizing load and work to puncture depend on precise thickness measurements, as cocoon segments may deviate from uniform thickness, potentially leading to over- or underestimation of the true puncture resistance properties values. While this protocol focuses on standardized cocoon segments for reproducible testing conditions, future studies could explore testing whole cocoons to evaluate puncture resistance properties under different testing setups.

**Figure 8:**
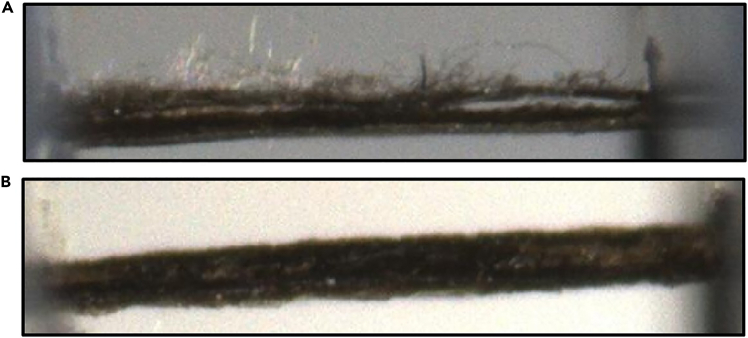
Screenshots of the MicroTester software assessing the cocoon segment condition assessment (A) Damaged cocoon segment with visible layer separation, likely due to a dull cutting tool. (B) Properly prepared segment with clean and visible edges suitable for thickness measurements.

**Figure 9:**
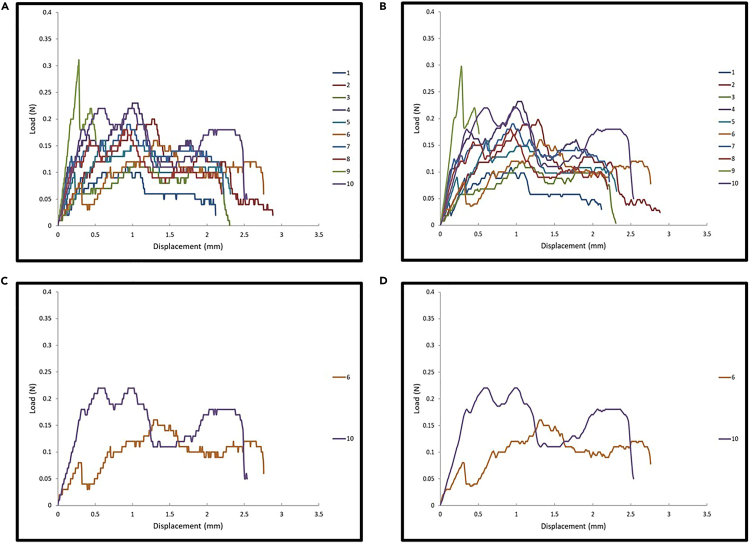
Effect of 5-point moving average on load (N) - displacement (mm) curve visualization (A and B) Complete dataset showing all puncture tests for female cocoons: (A) raw load and displacement data, (B) 5-point moving average applied. (C and D) Representative individual tests: (C) raw load and displacement data, (D) 5-point moving average applied. Note the noise reduction while preserving the key puncture resistance properties and trends observed along the test.

## Troubleshooting

### Problem 1

• Cocoon segment damage or mishandling during cutting using the 3D stencil and mounting procedures onto the testing 3D-printed fixtures (related to Steps 2–5, 9–19, 32–36).
• Cocoon segments may sustain damage at several points during the protocol, resulting from improper handling, mechanical damage during cocoon collection, or dull cutting tools. This can compromise data integrity and reduce the resulting sample sizes available for data and statistical analysis.

### Potential solution

• Inspect the cocoons and cocoon segments continuously during the experiment and maintain a set of additional cocoons that can be processed if needed to complete the intended data set.
• If a cocoon segment appears to be damaged during the thickness measurement procedure, discard and prepare new segments from the same cocoon if material is available.
• If a cocoon segment appears to be damaged during the puncture testing segments, discard both the damaged segment and its corresponding thickness measurement data to maintain paired data integrity across the experimental design.
• A side-by-side example of a damaged versus an adequately prepared cocoon segment can be found in Figure 8.
• Record the reason for exclusion in your data sheet and ensure adequate sample sizes are maintained across experimental groups.
• Continuously check the sharpness of the cutting tools and use gentle handling techniques to prevent damage during preparation.

### Problem 2

• Inconsistent thickness measurements (related to Steps 16–29).
• Thickness measurements may vary between measurement attempts on the same sample due to mechanical damage to the cocoons and segments, mishandling, storage conditions, and natural variation between cocoons and cocoon segments.

### Potential solution

• Establish a consistent preload methodology by gently handling the sample mounting.
• Inspect the cocoon segment under a light microscope at a higher magnification if suspected damage occurred.
• Ensure the specimen is positioned correctly, perpendicular to the Microtester camera.
• Evaluate if the settings in the MicroTester software need to be adjusted for a more precise visualization of the cocoon segment.

### Problem 3

• Cocoon segment sample mounting and adhesion issues (related to Steps 32–38).
• Cocoon segments may detach from C-cards during testing, resulting in invalid load (N) and displacement (mm) measurements and potential data point loss.

### Potential solution

• Consider using an alternative tape or superglue.
• Press the cocoon segment directly onto the C-card for 30 seconds to ensure complete contact.
• Evaluate the time allowed for superglue curing between preparation and testing.
• Assess if test parameter settings are consistent with previous samples. Higher cross-head speeds may cause superglue failure before cocoon puncture occurs.
• Consider alternative mounting methods, such as small clamps, alternative fixtures, or different adhesive systems, if the transparent tape and superglue combinations prove insufficient for your specimen type.

### Problem 4

• Load (N) and displacement (mm) curves show irregular patterns, excess noise, or pixelated curves (related to Steps 47–54).
• Load (N) and displacement (mm) data may contain noise due to instrument sensitivity, low load values depending on the type of sample, or high data acquisition frequency. The noise observed in the load (N) - displacement (mm) curves can interfere with the observation of deformation behavior and the analysis of load (N) and displacement (mm) trends between samples and test groups.

### Potential solution

• If the noise interferes with the data analysis, consider purchasing a smaller load cell for the testing instrument to bring the maximum load closer to the range of the samples.
• Apply a 5-point moving average to the load (N) and displacement (mm) values to reduce noise while preserving the overall curve characteristics, following established protocols.^8^
• After applying the 5-point moving average, identify the data starting point as discussed in the step-by-step method details section, then apply the zeroing formulas to the averaged load (N) and displacement (mm) data.
• Use the zeroed averaged data to generate load (N) - displacement (mm) curves and to calculate the other puncture resistance properties.
• An example of load (N) - displacement (mm) curves generated using the point-averaged method can be found in Figure 9.

### Problem 5

• Inconsistent needle penetration angle (related to Steps 34–39).
• Misalignment between the needle and cocoon segment centerline may result in oblique puncture angles, affecting recorded puncture resistance properties and resulting in non-representative failure during testing. This manifests as asymmetric puncture sites, irregular load (N) - displacement (mm) curves, or altered max puncture load values.

### Potential solution

• Verify needle alignment perpendicular to the specimen surface by adjusting the position of the top fixture and the position of the top fixture is gripped to the top clump before the start of each test.
• Confirm the C-card is mounted flat against the bottom fixture before mounting the 3D-printed fixture onto the MTS Synergie 100 mechanical testing instrument bottom clamp.
• Check for cocoon segment curling caused by incorrect stencil dimensions or cutting angles, which can elevate portions of the specimen and create non-perpendicular contact with the needle.
• Observed the needle to ensure it is not bent or damaged before each test.
• Consider replacing the 3D-printed fixture components if potential wear and tear persist despite adjustments.

## Resource availability

### Lead contact

Further information and requests for resources and reagents should be directed to and will be fulfilled by the lead contact, Oran Wasserman (oran.wasserman@usu.edu).

### Technical contact

Technical questions on executing this protocol should be directed to and will be answered by the technical contact, Dr. Justin A. Jones (justin.a.jones@usu.edu).

### Materials availability

STL files for all custom 3D-printed fixtures (cocoon cutting stencil, top fixture, and bottom fixture) are available in the supplemental materials (Data S1, S2, S3, S4, and S5). This study did not generate new, unique reagents.

### Data and code availability

This protocol does not generate datasets requiring public deposition. The raw data used to create the representative data and load (N)-displacement (mm) curves, and the data presented in the expected outcomes section, are available from the corresponding author on request.

## Acknowledgments

We want to thank Dr. Lindsie M. McCabe and Dr. Diana L. Cox-Foster at the USDA-ARS Pollinating Insects Research Unit, Logan, UT, for providing bees for this project. We also want to thank Ethon D. Van Noy for his contribution to the data analysis done for this protocol. This research received no specific grant from any funding agency in the public, commercial, or not-for-profit sectors. Figures that are part of the graphical abstract were created with BioRender. Williams, M-K. (2025) https://BioRender.com/6oi6w3p.

## Author contributions

Conceptualization, O.W., M.R.W., S.F., and J.A.J.; methodology, O.W., M.R.W., S.F., B.E.B., and M.-K.F.W.; software, O.W., M.R.W., B.E.B., and M.-K.F.W.; validation, O.W., M.R.W., and B.E.B.; formal analysis, O.W. and M.R.W.; investigation, O.W. and M.R.W.; resources, J.A.J.; data curation, O.W. and M.R.W.; writing – original draft, O.W. and M.R.W.; writing – review and editing, O.W., M.R.W., S.F., B.E.B., M.-K.F.W., and J.A.J.; visualization, O.W., M.R.W., S.F., B.E.B., and M.-K.F.W.; supervision, O.W. and J.A.J.; project administration, J.A.J.; funding acquisition, J.A.J.

## Declaration of interests

The authors declare no competing interests.

## Supplemental information

Document S1. Figures S1–S5

Data S1. STL file for the 3D-printed clamps

Data S2. STL file for the 3D-printed tower mount for thickness measurement

Data S3. STL file for the 3D-printed cutting stencil (7 mm × 2 mm)

Data S4. STL file for the 3D-printed bottom fixture with 6 mm × 5.5 mm opening

Data S5. STL file for the 3D-printed top fixture with Luer-Lock adapter
