# Supplementary material for: Protocol for quantifying the thickness and puncture resistance properties of solitary bee cocoons using Osmia lignaria as a model
Source: STAR Protoc. 2025 Nov 14;6(4):104191. doi: 10.1016/j.xpro.2025.104191 (PMC12664038; doi:10.1016/j.xpro.2025.104191)
Supplement: Document S1. Figures S1–S5 [file mmc1.pdf]

Supplemental Materials

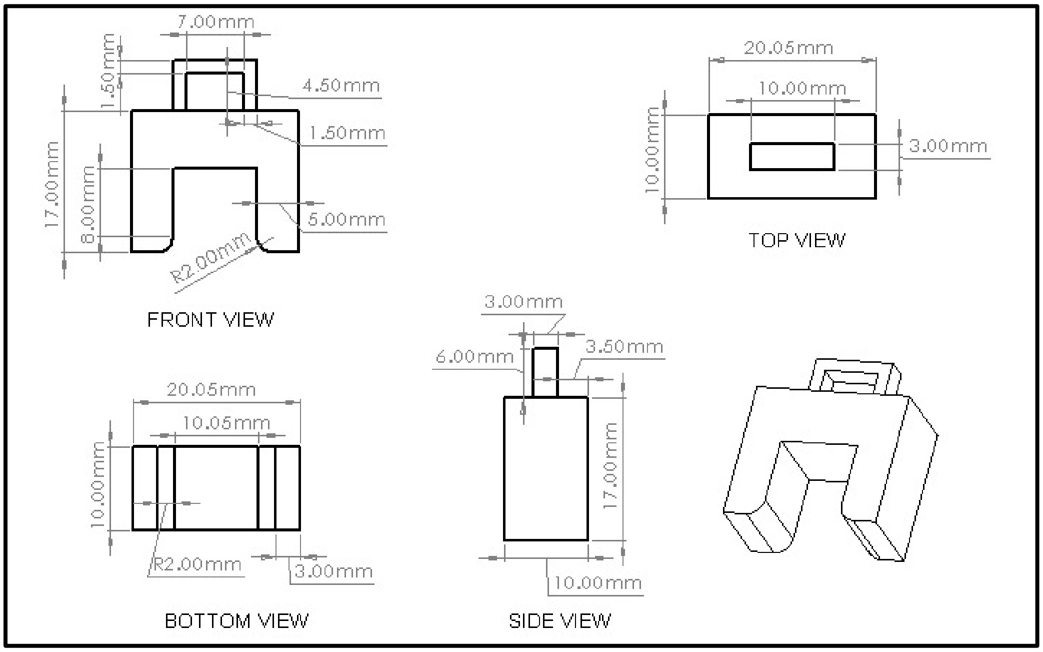

Figure S1. 3D-printed clamps designed in SolidWorks (2023) for securing cocoon segments to the tower mount during thickness measurements with the CellScale MicroTester, related to Step 5a in preparation two section.

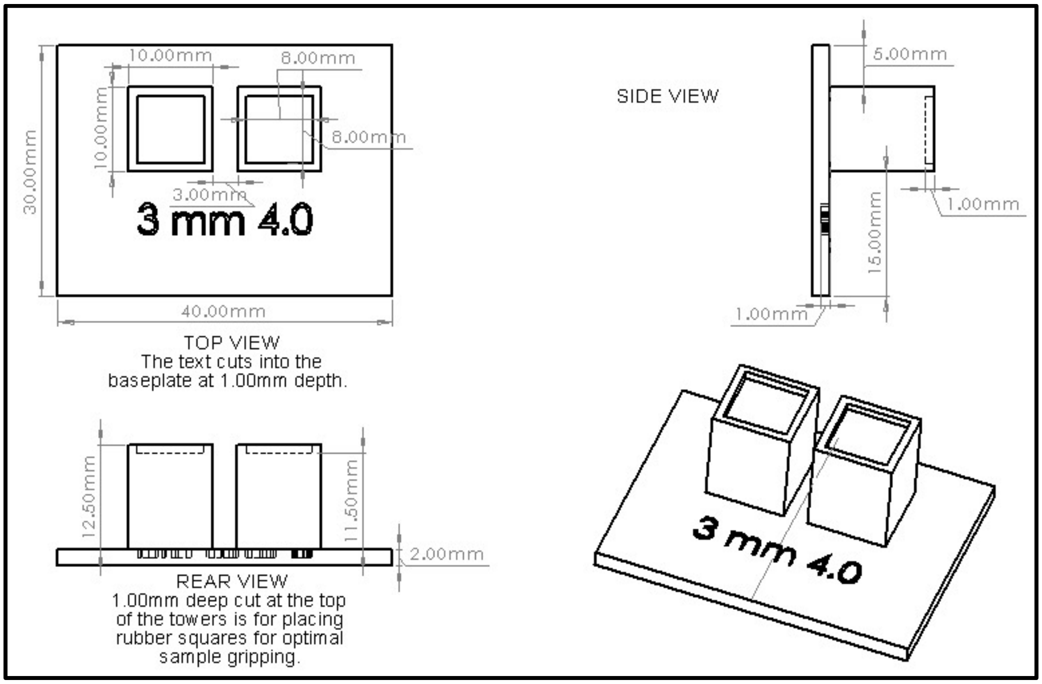

Figure S2. 3D-printed tower mount with 3 mm gap designed in SolidWorks (2023) for cocoon segment positioning during thickness measurements with CellScale MicroTester, related to Step 5b in preparation two section.

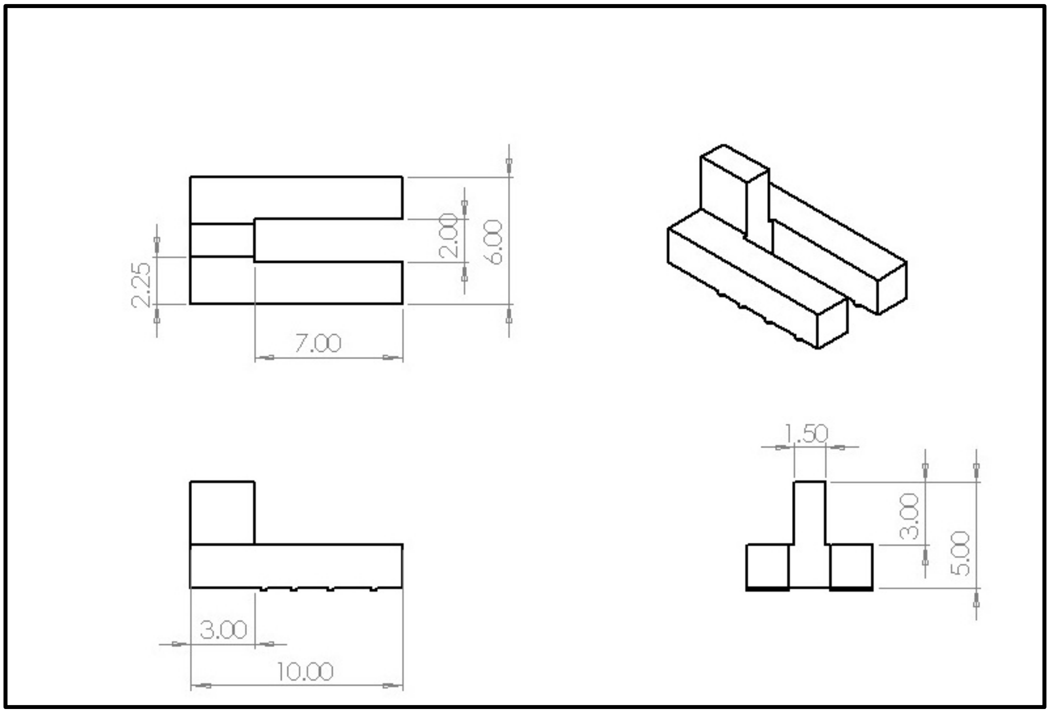

Figure S3. 3D-printed cutting stencil with 7 mm × 2 mm opening designed in SolidWorks (2023) for consistent cocoon segment preparation, related to Step 5c in preparation two section.

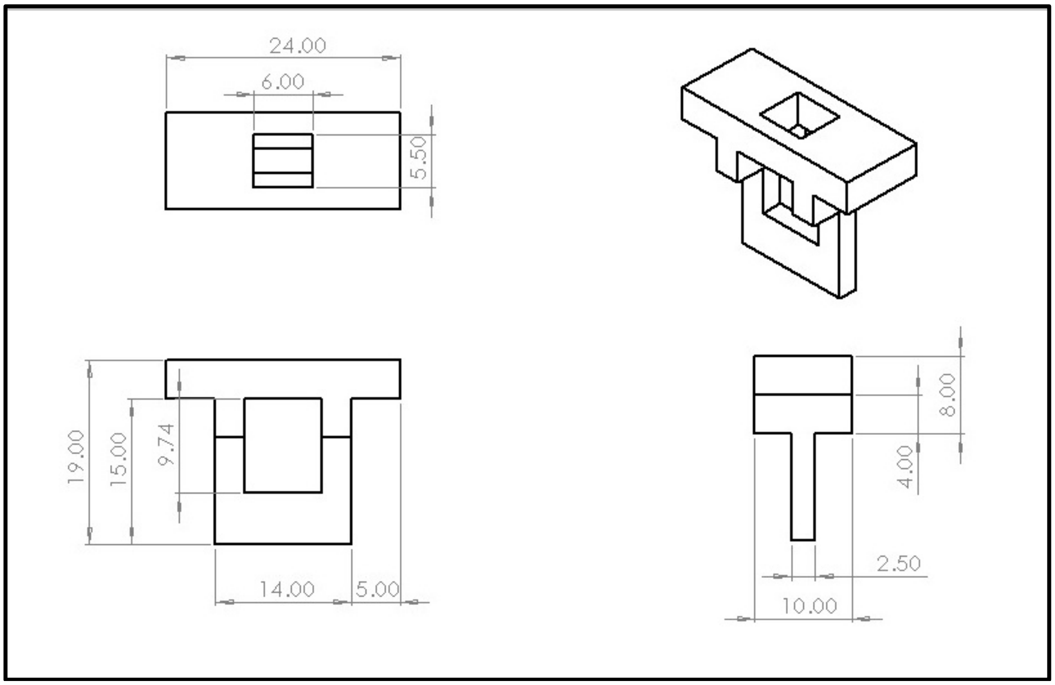

Figure S4. 3D-printed bottom fixture with 6 mm × 5.5 mm opening designed in SolidWorks (2023) for C-card mounting during puncture testing, related to Step 5d in preparation two section.

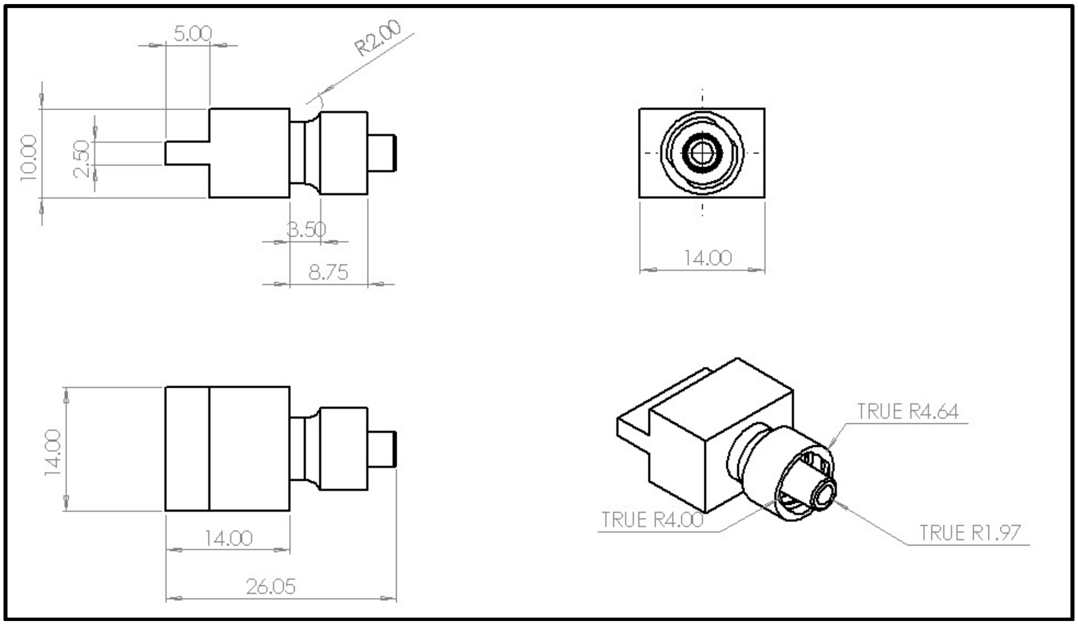

Figure S5. 3D-printed top fixture with integrated Luer-Lock adapter designed in SolidWorks (2023) for 27.5-gauge needle mounting during puncture testing, related to Step 5e in preparation two section.
